# Benefits of thermal imaging in orbital inflammation disorders

**DOI:** 10.1186/s12348-026-00577-y

**Published:** 2026-05-12

**Authors:** Clément Rocchi, Stephanie Telo, Nicolas Chevalier, Nathalie Tieulie, Lydiane Mondot, Albert Themelin, Stephanie Baillif, Arnaud Martel

**Affiliations:** 1 Ophthalmology Department, Pasteur 2, Nice University Hospital, 30 Voie Romaine, Nice, 06000 France; 2 Endocrinology Department, L’Archet, Nice University Hospital, 151 Route Saint-Antoine de Ginestière, Nice, 06202 France; 3 Rheumatology Medicine Department, Pasteur 2, Nice University Hospital, Côte d’Azur University, 30 Voie Romaine, Nice, 06002 France; 4 Radiology Department, Pasteur 2, University Hospital, UR2CA URRIS, Côte d’Azur University, 30 Voie Romaine, Nice, 06002 France

**Keywords:** Orbital inflammation, Thermal imaging, Thyroid eye disease, Clinical activity score

## Abstract

**Background/aims:**

Assessing periocular inflammation is essential in several disorders, including Thyroid Eye Disease (TED). The Clinical Activity Score (CAS) is the most commonly used method to assess orbital inflammation, but is associated with significant inter-observer variability. The aim of this study was to assess the ability of Infrared Thermography (IRT) to detect orbital inflammation in TED and other orbital inflammation disorders (OOID).

**Methods:**

A retrospective study was conducted between March 2020 and November 2023. Patients were divided into four groups: active TED (CAS ≥ 3), non-active TED (CAS < 3), OOID, and healthy controls. Demographics, proptosis, and CAS were recorded. IRT was performed in 6 periocular areas, including the caruncle. Four IRT periocular patterns were characterized.

**Results:**

Hundred and ten patients (63.64% of women) with a mean age of 59.47 (25–93) years were included. Thirteen (11.82%) patients were included in the active TED group, 44 (40.00%) in the non-active TED group, 17 (15.45%) in the OOID group and 36 (32.73%) in the control group. Non-active TED and control patients had lower mean caruncular and periocular temperatures compared to active TED and OOID patients (*p* < 0.05). The caruncular temperature was significantly higher in active TED patients compared to OOID patients (*p* < 0.05). Non-active TED and control patients mainly showed round and upper coma IRT patterns while active TED and OOID patients showed crab claw and other IRT patterns (*p* < 0.05).

**Conclusion:**

Periocular IRT measurement is a rapid, simple, non-invasive, cost-effective, and reproducible method for detecting orbital inflammation and allows differentiating active TED from OOID.

## Introduction

Orbital inflammation disorders are commonly found in daily clinical practice and may be divided into specific or non-specific disorders [1]. Among them, thyroid eye disease (TED) is the most common specific orbital inflammation disorder and is also the most common extra-thyroid manifestation of Grave’s disease with an annual incidence of 20–50 per 100,000 population [2, 3]. TED is characterized by an enlargement of the periocular fat and extraocular muscles resulting in various clinical signs such as proptosis, lid retraction and strabismus. TED has been shown to negatively impact patients‘ quality of life [4–6]. TED follows a biphasic course, also called Rundle’s curve, with an active inflammatory phase followed by an inactive fibrotic phase [7]. When clinical signs of orbital inflammation are present, anti-inflammatory drugs such as intravenous steroids, anti-IL-6, anti-CD20 or more recently anti-Insulin Growth Factor Receptor 1 (IGF-1R) may be prescribed to reduce orbital inflammation and TED-related complications [8].

In 1989, Mourits and coworkers have developed the Clinical Activity Score (CAS) with the aim to assess whether anti-inflammatory drugs should be prescribed or not [9, 10]. The CAS is a 7-item scale assessing periocular pain, eyelid swelling and redness, conjunctiva swelling (chemosis) and redness as well as caruncular inflammation. A CAS ≥ 3 is an indication for prescribing anti-inflammatory drugs. The CAS can be easily and rapidly assessed by any practitioner evaluating a TED patient, including ophthalmologists, endocrinologists, and nuclear medicine practitioners, and no specific material is required. However, the CAS is poorly reproducible from one assessor to another. A recent study has shown that the interobserver variability in CAS between 6 experienced observers was not reliable [11]. Such a variability may lead to overtreatment of TED patients. There is therefore a need to improve the assessment of orbital inflammation in TED and other orbital inflammatory disorders (OOID).

Magnetic resonance imaging (MRI) has proven to be a reliable tool for assessing several components of orbital inflammation, but it is costly, poorly available in several regions, not suitable for claustrophobic patients and needs to be repeated over time [12, 13]. Infrared Thermography (IRT) is a simple, fast, non-invasive, and easily reproducible tool for assessing skin temperature by capturing infrared radiations. Several studies have shown the value of IRT for detecting inflammation in several ocular surface disorders [14–16]. Several authors have assessed the use of IRT in TED and found a direct correlation with the CAS, the presence of proptosis and the level of anti-thyroid peroxidase antibodies [17–22]. However, these studies have several limitations. First, the authors have only investigated numerical skin temperature values that significantly differ from one patient to another without clearly defining a skin temperature “cut-off”, making their interpretation difficult in daily clinical practice. Second, no study has investigated the value of IRT in other periocular inflammation disorders.

The aim of this study was to investigate the value of IRT in TED and OOID patients using simple, reliable measurements that could be used in daily clinical practice.

## Patients and method

### Study design

A retrospective study was conducted in Nice University Hospital, France between March 2020 and November 2023. All patients gave oral consent for this study as well as for data storage, including clinical photos. This study was approved by the French Ophthalmic Society (Number: IRB 00008855).

### Study participants

Participants were divided into 4 groups: the active TED group, including patients with TED with a CAS ≥ 3; the non-active TED group, including patients with TED with a CAS < 3; the OOID group, including patients with specific or non-specific orbital inflammation syndrome, excluding TED and regardless of the site of inflammation (extraocular muscle, lacrimal gland, orbital fat). In this group, the CAS was also used to assess orbital inflammation symptoms; and the control group, including healthy subjects without any ophthalmological disorder.

### Data collected

The following data were collected: demographics and clinical data, including the age, gender, past medical or surgical history and smoking status. For TED patients, the disease duration, current medical treatment, past radical treatment (i.e., thyroidectomy or radioactive iodine) and last TRAb (thyrotropin receptor antibody) level were also collected.

Patients underwent an ophthalmological examination, including proptosis measurement with Hertel exophthalmometer, diplopia assessed using the Gorman score and orbital inflammation assessed using the 7-item CAS.

**Fig. 1:**
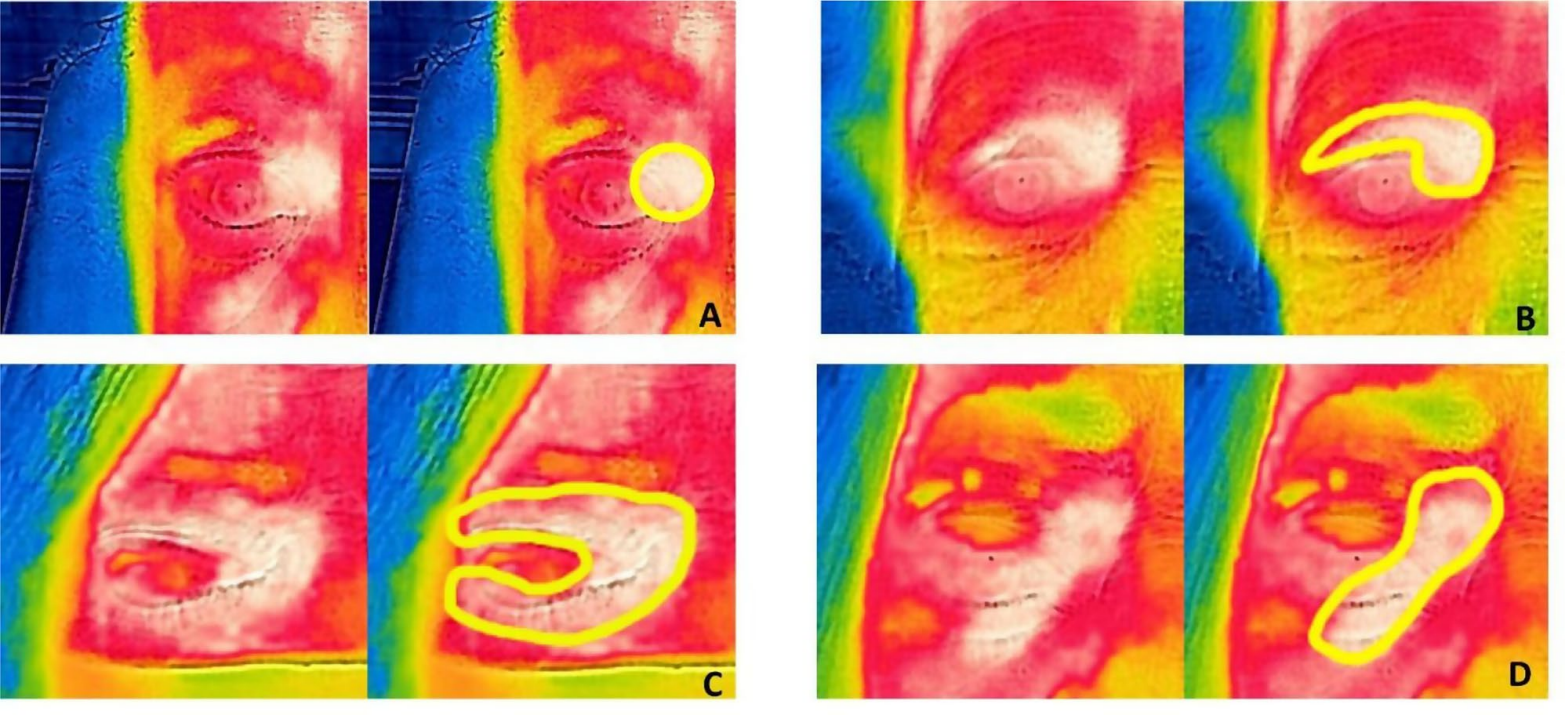
Thermal imaging patterns (round-shape pattern, upper coma pattern, crab claw pattern, other patterns [lower coma, frontal diffusion, external islet])

IRT temperatures were measured using a FLIR C3 camera (FLIR Systems, Inc., Wilsonville, Oregon USA) and analyzed using the FLIR Tools application. Measurements were taken in a quiet, temperature-controlled room maintained at 24 °C and after at least 5 min of rest, usually at the end of the consultation. The following areas were systematically recorded: forehead and lateral orbit (reference temperatures), caruncular area, and the hottest and coldest periocular areas determined automatically by the camera. Other measurements were made using the FLIR Tools application, which allowed us to analyze and manage the data. Specifically, the mean periocular temperature was calculated by creating a rectangular zone that encompassed the eye, the upper and lower eyelids. Several ratios were calculated based on the reference temperatures to better take into account the inter-individual body temperature. For example, the following ratios were calculated: $\frac{Hottest\,spot}{Lateral\,orbit\,temperature}$ and $\frac{Hottest\,spot}{Forehead\,temperature}$. Various IRT patterns were also identified to facilitate IRT interpretation in daily clinical practice. Based on our experience, patients were classified into four IRT pattern groups: (i) round shape, (ii) upper coma, (iii) crab claw, and (iv) other patterns (frontal diffusion, inferior coma, external islet) (Fig. 1). Only patients with active TED underwent a thorough follow-up re-evaluation with IRT measurements to assess the efficacy of anti-inflammatory treatment.

Only one analysis was performed for each patient: in the active TED group, the eye showing the higher CAS was analyzed. In the OOID group, only the eye affected by orbital inflammation was analyzed. In the non-active TED and control groups, we decided to analyse the eye with the hottest spot showing the higher IRT value. All clinical and IRT measurements were assessed by the same operator (AM).

### Statistical analysis

Numerical variables are presented as mean ± SD and categorical variables as absolute and relative (%) frequencies. Group comparability was assessed by comparing baseline demographics and follow-up duration across groups. Normality and heteroscedasticity of continuous variables were assessed using the Shapiro–Wilk test and Levene’s test, respectively. Continuous outcomes were compared using one-way ANOVA, Welch’s ANOVA, or the Kruskal–Wallis test depending on data distribution and variance equality. Categorical outcomes were compared using the chi-squared test or Fisher’s exact test, as appropriate. All tests were two-tailed and the significance level was set at α = 0.05.

Given the small sample size of the active TED group (*n* = 13), pairwise comparisons between active and non-active TED for caruncle and periocular temperatures were additionally assessed using the Mann–Whitney U test, and value distributions were displayed using box plots.

Caruncular temperature and CAS were assessed for normality using the Shapiro–Wilk test. The association between caruncular temperature and CAS was evaluated using Spearman’s rank correlation coefficient. Statistical analyses were performed using EasyMedStat (version 3.35; www.easymedstat.com*).*

## Results

Overall, 110 patients (63.64% of women) with a mean age of 59.47 (25–93) years were included in the study. Demographics are presented in Table 1 according to the group.

**Table 1:** Baseline patients’ characteristics

Patient characteristics	Active TED (*n* = 13)	Non-active TED (*n* = 44)	OOID (*n* = 17)	Controls (*n* = 36)	*P*-value
Gender, n (%)					0.08
Men	3 (23.08)	12 (27.27)	6 (35.29)	19 (52.78)	
Women	10 (76.92)	32 (72.73)	11 (64.71)	17 (47.22)	
Mean age ± SD, years	55.31 ± 16.15	52.64 ± 15.95	62.94 ± 15.27	67.00 ± 20.78	< 0.05
Comorbidities: n (%)					
Chronic disease	4 (30.77)	10 (22.73)	5 (29.41)	13 (36.11)	0.63
Other auto-Immune diseases	2 (15.38)	9 (20.45)	2 (11.76)	4 (11.11)	0.71
Mean thyroid disease duration ± SD, months	90.05 ± 154.62	61.83 ± 114.01	NA	NA	0.23
Mean CAS ± SD					
Right Eye	3.54 ± 0.97	0.27 ± 0.54	0	0	< 0.05
Left Eye	3.54 ± 1.90	0.29 ± 0.55	0.28 ± 0.76	0	< 0.05
Mean TRAb level ± SD, IU/mL	13.32 ± 12.32	22.23 ± 24.31	NA	NA	0.50
Smoking status: n (%)	5 (38.46)	16 (36.36)	2 (11.76)	5 (13.88)	< 0.05
Thyroidectomy: n (%)	0	3 (6.82)	0	0	0.42
Radioiodine treatment: n (%)	0	4 (9.09)	0	0	0.21
Patients taking anti-thyroid drugs: n (%)	12 (92.31)	28 (65.12)	0	0	< 0.05
Patients taking thyroid hormone treatment: n (%)	8 (61.54)	24 (55.81)	0	0	< 0.05
Patients taking β-blockers: n (%)	2 (15.38)	8 (18.18)	0	0	< 0.05
Proptosis: n (%)	12 (92.31)	36 (81.82)	8 (61.54)	0	0.18
Mean proptosis ± SD in the right eye, mm	25.31 ± 4.40	22.08 ± 2.85	19.5 ± 4.06		< 0.05
Mean proptosis ± SD in the left eye, mm	24.92 ± 3.59	21.86 ± 3.05	20.3 ± 3.83		< 0.05
Diplopia: n (%)	5 (38.46)	13 (29.55)	0	0	0.09

OOID: other orbital inflammatory disorders; NA: not available; CAS: clinical activity score; SD: standard deviation; TED: thyroid eye disease; TRAb: thyroid-stimulating hormone receptor antibodies

Thirteen patients (11.82%) were included in the active TED group, 44 (40.00%) in the non-active TED group, 17 (15.45%) in the OOID group and 36 (32.73%) in the control group. In the OOID group, 5 patients (29.41%) had dacryoadenitis, 4 patients (23.53%) had myositis, and 8 patients (47.06%) had diffuse inflammation. In the dacryoadenitis subgroup, the disorder was always unilateral, and the etiologies included 2 cases of sarcoidosis, 1 case of Gougerot-Sjögren syndrome, 1 case of IgG4-related disease, and 1 idiopathic case. In the myositis subgroup, there were 1 case of granulomatosis with polyangiitis, 1 post-viral case (COVID-19), and 2 idiopathic cases. In the diffuse inflammation subgroup, there were 4 cases of arteriovenous fistula, 3 cases of idiopathic diffuse inflammation, and 1 case of IgG4-related disease. Patients in the active and non-active TED groups were significantly younger and more likely to smoke (*p* < 0.05), regardless of the CAS. Patients in the active TED group had a significantly higher CAS and were more likely to have proptosis at baseline (*p* < 0.05).

Tables 2 and 3 summarize the IRT measurements according to the group.

**Table 2:** Thermal imaging findings according to the group

	Active TED (*n* = 13)	Non-active TED (*n* = 44)	OOID (*n* = 17)	Controls (*n* = 36)	*P*-value
Mean forehead temperature ± SD, °C	34.44 ± 0.68	33.83 ± 0.95	34.16 ± 0.75	33.49 ± 0.89	< 0.05
Mean lateral orbit temperature ± SD, °C	33.67 ± 1.00	33.16 ± 0.80	33.90 ± 0.79	33.44 ± 0.84	**< 0.05**
Mean hottest spot temperature ± SD, °C	36.29 ± 1.01	35.69 ± 0.60	35.91 ± 0.45	35.71 ± 0.56	0.24
Mean hottest spot temperature/forehead temperature ratio	1.05	1.06	1.05	1.07	**< 0.05**
Mean hottest spot temperature/lateral orbit temperature ratio	1.08	1.08	1.06	1.07	0.08
Location of the hottest spot: n (%)					**< 0.05**
superomedial	12 (92.31)	43 (97.73)	13 (76.47)	36 (100)	
superolateral	1 (7.69)	0	3 (17.65)	0	
inferomedial	0	1 (2.27)	1 (5.88)	0	
inferolateral	0	0	0	0	
cornea	0	0	0	0	
Mean coldest spot temperature ± SD, °C	32.39 ± 1.52	31.82 ± 0.97	32.45 ± 1.10	31.79 ± 0.91	0.06
Location of the coldest spot: n (%)					**< 0.05**
superomedial	0	0	0	0	
superolateral	8 (61.54)	38 (86.36)	12 (70.59)	28 (77.78)	
inferomedial	3 (23.08)	1 (2.27)	4 (23.53)	0	
inferolateral	2 (15.38)	4 (9.09)	1 (5.88)	8 (22.22)	
cornea	0	1 (2.27)	0	0	
Mean caruncle temperature ± SD, °C	35.76 ± 0.94	34.63 ± 0.77	35.05 ± 0.56	34.52 ± 0.67	**< 0.05**
Mean periocular area temperature ± SD, °C	34.79 ± 1.1	34.09 ± 0.71	34.59 ± 0.54	34.03 ± 0.70	**< 0.05**
Thermal Imaging Patterns: n (%)					**< 0.05**
round shape	2 (15.38)	21 (47.73)	3 (17.65)	11 (30.56)	
upper comma	0 (0.0)	20 (45.45)	4 (23.53)	25 (69.44)	
crab claw	7 (53.85)	1 (2.27)	7 (41.18)	0	
other patterns	4 (30.77)	2 (4.55)	3 (17.65)	0	

OOID: other orbital inflammatory disorders; SD: standard deviation; TED: thyroid eye disease

**Table 3:** Group comparison with p-values

	Active vs. non-active TED	Active TED vs. OOID	Active TED vs. Controls	Non-active TED vs. OOID	Non-active TED vs. controls	OOID vs. controls
Forehead (°C)	0.23	0.60	< 0.05 *	0.349	< 0.05 *	< 0.05 *
Lateral orbit (°C)	0.06	0.49	0.65	**< 0.05** *	0.11	0.09
Hottest Spot (°C)	0.13	0.90	0.12	0.168	0.89	0.21
Location of the hottest spot	0.41	0.78	0.26	**< 0.05** #	> 0.99	**< 0.05** #
Coldest Spot (°C)	0.11	0.90	**< 0.05** *	**< 0.05** *	0.92	**< 0.05** *
Location of the coldest spot	**< 0.05** #	0.85	**< 0.05** #	**< 0.05** #	0.21	**< 0.05** #
Caruncle (°C)	**< 0.05** *	**< 0.05** *	**< 0.05** *	0.07	0.38	**< 0.05** *
Periocular area (°C)	< 0.05 *	0.52	**< 0.05** *	**< 0.05** *	0.84	**< 0.05** *
Pattern	**< 0.05** #	0.28	**< 0.05** #	**< 0.05** #	0.07	**< 0.05** #

OOID: other orbital inflammatory disorders; TED: thyroid eye disease; (*) Student’s T-test; (#) Fisher’s exact test

No differences in the mean hottest spot were found between the groups. The coldest spots in the control and non-active TED groups were mainly located superolaterally compared to the active TED and OOID groups.

**Fig. 2:**
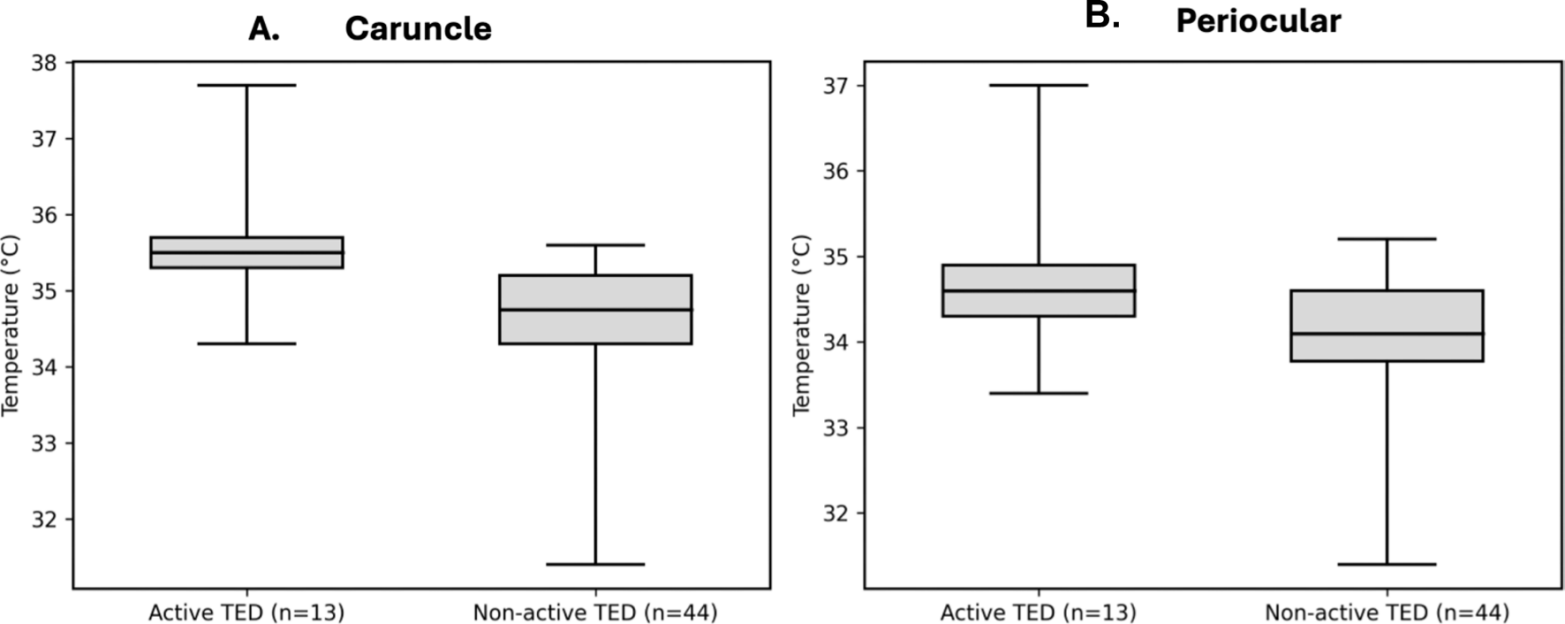
Box plots of caruncle temperature (**A**) and periocular temperature (**B**) in **active** versus **non-active** TED. (TED: thyroid eye disease)

The mean caruncular and periocular temperatures were lower in the non-active TED and control groups than in the active TED and OOID groups. When focusing on the comparison between active and non-active TED, caruncular temperature was significantly higher in active TED (Mann–Whitney U test, *p* = 0.00011), and periocular temperature (mean of upper and lower eyelid temperatures) was also higher in active TED with a borderline yet significant difference (Mann–Whitney U test, *p* = 0.046). The distributions of these two variables are presented using box plots (Fig. 2). The mean caruncular temperature, but not the mean periocular temperature, was significantly higher in the active TED group compared to the OOID group (*p* < 0.05).

Clinical features of the caruncle and conjunctiva were retrospectively assessed on baseline photographs. Carunculitis and chemosis were recorded as binary variables (present/absent). Carunculitis was more frequent in active TED than in non-active TED (6/13 [46.2%] vs. 2/44 [4.5%]; Fisher’s exact test, *p* = 0.0010; OR 18.0, 95% CI 3.01–107.72). Chemosis was also more frequent in active TED (7/13 [53.8%] vs. 5/44 [11.4%]; Fisher’s exact test, *p* = 0.0029; OR 9.1, 95% CI 2.17–38.17) (Table 4). These findings support the higher temperatures observed at the caruncle and in the periocular region in active TED.

**Table 4:** Clinical caruncle and conjunctival findings in active vs. non-active TED

	Active TED (*n* = 13)	Non-active TED (*n* = 44)	OR (95% CI)	*p*-value
Carunculitis	6 (46.2%)	2 (4.5%)	18.0 (3.0-107.7)	< 0.005
Chemosis	7 (53.8%)	5 (11.4%)	9.1 (2.2–38.2)	**< 0.005**

Significant differences in IRT patterns were found between the groups. In the non-active TED and control groups, round and upper coma patterns were the most common whereas in the active TED and OOID groups, crab claw and other patterns were the most common (*p* < 0.05). The IRT patterns did not allow differentiating active TED from OOID (*p* = 0.28).

Among the 13 active TED patients included, 9 were followed after receiving anti-inflammatory treatment (Table 5).

**Table 5:** Thermal imaging changes in patients with active thyroid eye disease between before and after systemic anti-inflammatory treatment. Five patients were treated with IV steroids and 4 patients with IV tocilizumab

	Before treatment (*n* = 13)	After treatment (*n* = 9)	*p*-value
Mean CAS ± SD			
Right Eye	3.54 ± 0.97	1.0 ± 0.87	**< 0.05**
Left Eye	3.54 ± 1.90	0.667 ± 0.87	**< 0.05**
Mean proptosis size ± SD, mm			
Right Eye	25.31 ± 4.40	24.06 ± 2.96	0.61
Left Eye	24.92 ± 3.59	23.0 ± 2.06	0.13
Mean TRAb level ± SD, IU/mL	13.32 ± 12.32	3.72 ± 2.68	0.09
Mean forehead temperature ± SD, °C	34.44 ± 0.68	33.66 ± 1.09	0.06
Mean lateral orbital temperature ± SD, °C	33.67 ± 1.00	33.27 ± 1.29	0.84
Mean hottest spot temperature ± SD, °C	36.29 ± 1.01	35.57 ± 0.85	0.18
Location of the hottest spot: n (%)			> 0.99
superomedial	12 (92.31)	9 (100)	
superolateral	1 (7.69)	0	
inferomedial	0	0	
inferolateral	0	0	
cornea	0	0	
Mean coldest spot ± SD, °C SD	32.39 ± 1.52	31.83 ± 0.64	0.31
Location of the coldest spot: n (%)			0.15
superomedial	0	1 (11.11)	
superolateral	8 (61.54)	8 (88.89)	
inferomedial	3 (23.08)	0	
inferolateral	2 (15.38)	0	
cornea	0	0	
Mean caruncle temperature ± SD, °C	35.76 ± 0.94	34.82 ± 1.01	**< 0.05**
Mean periocular area temperature ± SD, °C	34.79 ± 1.10	34.31 ± 0.90	0.29
Pattern: n (%)			**< 0.05**
Round shape	2 (15.38)	6 (66.67)	
Upper Coma	0 (0.0)	2 (22.22)	
Crab Claw	7 (53.85)	1 (11.11)	
Other Patterns*	4 (30.77)	0	

CAS: clinical activity score; SD: standard deviation; TRAb: thyroid-stimulating hormone receptor antibodies

**Fig. 3:**
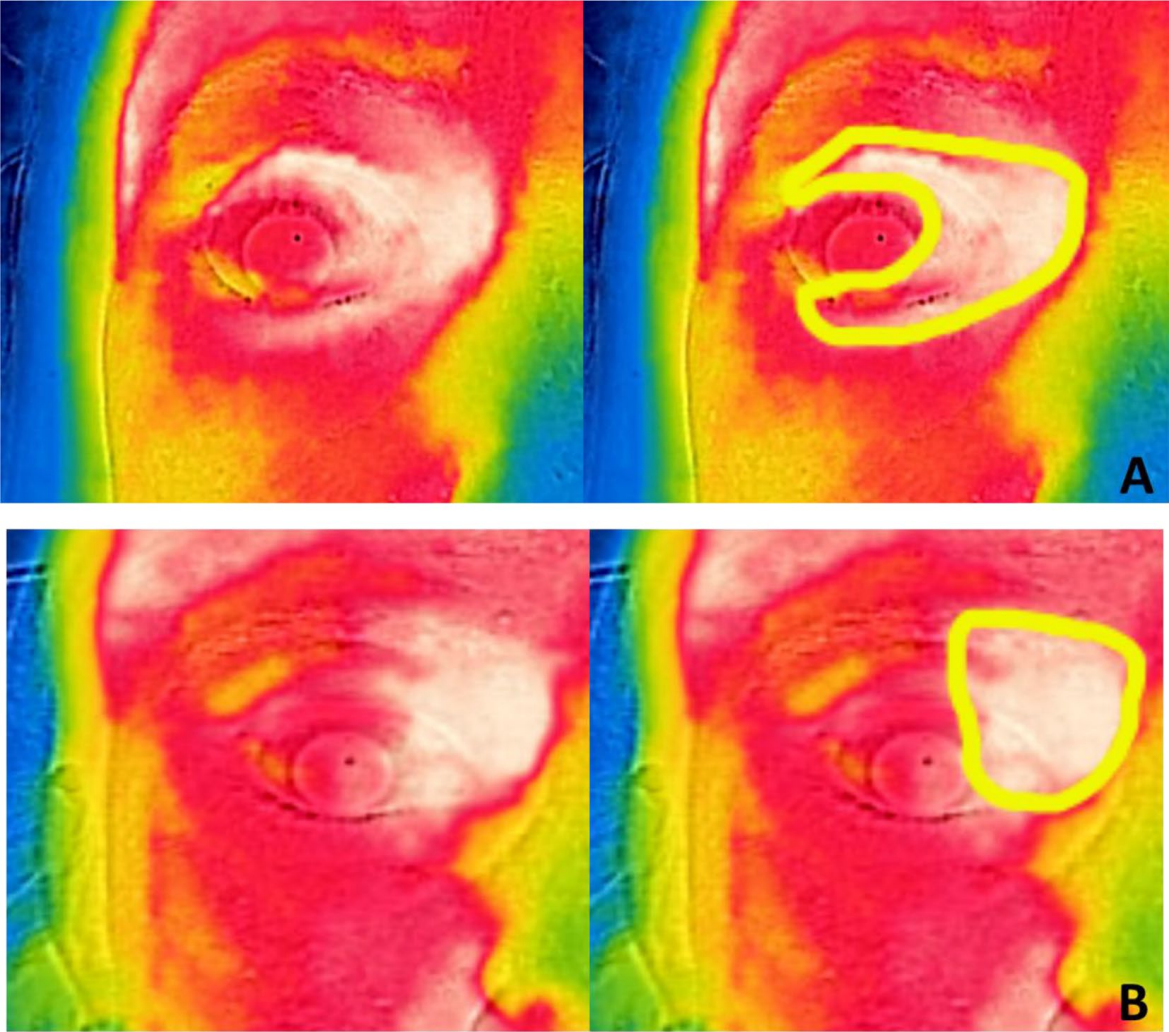
Changes in thermal imaging patterns between before and after treatment in patients with active TED. **A**: before treatment: crab claw pattern; **B**: after 12 boluses of corticosteroids: round-shape pattern

The follow-up period ranged from 2 to 13 months, depending on the case. Of these 9 patients, 5 were treated with IV steroids, and 4 with IV tocilizumab. The mean CAS and mean caruncular temperature were significantly reduced after treatment (*p* < 0.05). Post-treatment, active TED patients exhibited significant changes in IRT patterns, shifting from crab claw and other abnormal patterns to more physiological patterns, such as round-shape and upper coma, similar to those observed in control and non-active TED patients. These pattern changes remained stable as long as the orbitopathy remained non-active. (Fig. 3)

A low positive correlation was found between the CAS and the caruncular temperature in the active and non-active TED groups (ρ = 0.39; r2 = 0.151; *p* = 0.003).

## Discussion

This study confirmed that periocular IRT allows measuring rapidly, non-invasively, and reproducibly orbital inflammation. Our findings provided valuable insights into the thermal profiles associated with different orbital conditions and the identification of IRT patterns allowed performing a rapid assessment that is feasible in daily clinical practice. Only the assessment of the caruncular temperature allowed differentiating active TED from OOID patients.

The CAS is considered the gold standard for differentiating active from non-active TED but its subjectivity and lack of reproducibility from one practitioner to another are widely acknowledged [11]. The CAS allows determining whether systemic anti-inflammatory treatments, with their known side effects, are necessary or not [23]. This poor reproducibility of the CAS explains the development of alternative measurements of orbital inflammation through the use of various imaging modalities, including MRI and IRT.

In our study, we found that the location, but not the temperature, of the hottest and coldest IRT spots could be predictive of orbital inflammation. Under physiological conditions, the hottest IRT spot is usually located superomedially due to the presence of the supratrochlear and supraorbital neurovascular bundles whereas the coldest spot is usually located superolaterally [24]. Our results confirmed the superomedial location of the hottest IRT spot, except in the OOID group, where it was more often superolateral. This could be due to the fact that about one third of the patients in the OOID group had dacryoadenitis. Similarly, the loss of the superolateral position of the coldest spot was highly suggestive of orbital inflammation (active TED and OOID groups). The caruncular temperature was also associated with the presence of orbital inflammation and even allowed differentiating active TED from OOID. Interestingly, the caruncular temperature did not correlate with the CAS in the Spearman correlation analysis. No clear conclusion could be drawn from this result because the CAS is almost imperfect which justified this study. This could indicate a stronger discriminatory power of caruncular IRT compared to the subjective CAS. Further studies including other imaging modalities, such as MRI, are needed to answer this question. Although promising, IRT temperature measurements are not routinely used because no threshold has been defined. Using IRT patterns as described in our study is simpler and visually more meaningful than numerical temperature values, and they can be used in daily practice. We found that patients with orbital inflammation disorders (active TED and OOID) showed different patterns (crab claw and other patterns) compared to non-inflammatory patients who showed more physiological patterns (round shape and upper coma). A limitation of the IRT patterns is that they did not allow differentiating active TED from OOID unlike the measurement of the caruncular temperature. Finally, we also demonstrated the ability of IRT to assess the outcomes of anti-inflammatory treatments although only 9 patients were assessed.

Table 6 summarizes the published IRT studies conducted in TED patients. In line with previous studies, we found that the caruncular temperature was higher in active TED than in inactive TED. All the studies have reported a decrease in caruncular temperature after treatment.

**Table 6:** Literature review on previous thermal imaging studies in thyroid eye disease (TED) patients

	Number of eyes with active TED	Number of eyes with non-active TED	Number of healthy eyes	Mean age (years)	Mean CAS in active TED patients	Comparison of mean caruncle temperature in °C between active TED and non-active TED patients	Comparison of mean periocular area temperature in °C between active TED and non-active TED patients	Mean caruncle temperature in °C after treatment in active TED patients
Present study (2024)	13	44	36	59.50	3.54	35.75 vs. 34.63 (*p* < 0.05)	34.79 vs. 34.09 (*p* < 0.05)	34.82 (*p* < 0.05)
Prpic and al (2023)	29	21	0	58.24	3.41	36.35 vs. 35.10 (*p* < 0.05)	36 vs. 34.70 (*p* < 0.05)	NA
Dave and al (2021)	11	46	20	41.30	NA	35.92 vs. 34.84 (*p* < 0.05)	34.64 vs. 33.85	NA
Riguetto and al (2019)	12	62	62	49.60	4	38.40 vs. 36.13 (*p* < 0.05)	38 vs. 36.05	36.58 (*p* < 0.05)
Shih and al (2010)	46		14	46	3.06	33.56	32.65 (all patients)	33.30 (*p* < 0.05)
Chang and al (2006)	14		16	36.70	2.50	34.50 vs. 33 (*p* < 0.05)	32.57 (all patients)	33.60 (*p* < 0.05)

NA: not available; CAS: clinical activity score

This study has several strengths. First, our study is the first to include OOID patients. Interestingly, the caruncular temperature was significantly lower in OOID compared to active TED patients which could help clinicians to differentiate these two entities. Second, our study highlighted the need to develop more practical and easier-to-use IRT measurements in daily clinical practice. In addition to classical temperature measurements, we investigated the location of the hottest and coldest periocular spots that were easily identified using our IRT camera. We found that the loss of the superomedial location of the hottest spot as well as the loss of the superotemporal location of the coldest spot were highly suggestive of underlying orbital inflammation, without allowing differentiating active TED from OOID. We also described four different periocular IRT patterns that are easily identifiable in daily clinical practice. We found that non-inflammatory patients (non-active TED and healthy controls) had round-shape and upper coma patterns, while inflammatory patients showed different patterns (crab claw and other patterns). However, the periocular IRT patterns did not allow differentiating active TED from OOID.

However, several limitations must be stressed. First, the small number of active TED and OOID patients limited the statistical power of the study and did not allow performing multivariate analyzes. Larger studies are needed to confirm our findings and further prospective IRT studies could be conducted to investigate the efficacy of new anti-inflammatory drugs in active TED or OOID. Second, we did not investigate other imaging parameters such as MRI findings in this study. Given that the CAS is widely imperfect, visualizing orbital inflammation signs on MRI would have been pertinent. However, only a few non-active TED patients and none of the control patients underwent dedicated MRI. Further studies comparing IRT and MRI findings would thus be relevant.

## Conclusion

Our study confirmed the ability of periocular IRT imaging to detect orbital inflammatory disorders. The assessment of the caruncular temperature allowed differentiating active TED from OOID. The development of IRT patterns even allowed more rapidly and easily assessing the presence of orbital inflammation. Periocular IRT measurements appear as a rapid, easy, reproducible, non-invasive, and cost-effective method for assessing orbital inflammation and could reduce the inter-observer variability and subjectivity reported with the CAS. Further studies comparing IRT and MRI findings would be relevant to confirm this assumption.

## Data Availability

The datasets generated and/or analyzed during the current study are available from the corresponding author upon reasonable request.
